# Periumbilical perforating pseudoxanthoma elasticum

**DOI:** 10.1016/j.jdcr.2025.02.006

**Published:** 2025-03-04

**Authors:** Ana L. Duarte-Summers, Anna Chen, Morgan E. Sussman-McCrea, Shayan Waseh, Jason B. Lee, Sylvia Hsu

**Affiliations:** a Department of Dermatology, Temple University Lewis Katz School of Medicine, Philadelphia, Pennsylvania; b Department of Dermatology and Cutaneous Biology, Thomas Jefferson University, Philadelphia, Pennsylvania

**Keywords:** periumbilical perforating pseudoxanthoma elasticum, pseudoxanthoma elasticum

## Introduction

Pseudoxanthoma elasticum (PXE) is a heritable disorder of connective tissue, characterized by abnormal mineralization and progressive calcification of elastic fibers in various tissues, including the skin, retina, and cardiovascular system. The primary clinical features of cutaneous PXE typically manifest as coalescing yellowish papules on flexural areas of the skin. The pathogenesis of PXE is complex and remains unclear, though it has been linked to mutations in the *ABCC6* gene, which is involved in the regulation of extracellular matrix calcification and elastic fiber maintenance.^1^^,^^2^

Periumbilical perforating PXE (PPPXE) is a rare, localized variant of PXE, primarily seen in multiparous women, although cases in nonmultiparous individuals have also been reported.^3^ This variant presents with perforating lesions and yellow papules confined to the periumbilical region, often with no associated systemic findings characteristic of classical PXE.^4^ Several theories have been put forth to explain the etiology of PPPXE. The term was first used in 1979 when Hicks et al described 6 cases of multiparous women with PXE localized to the periumbilical region with no other features of classic PXE, initially proposing that PPPXE may be a separate entity.^4^ The rare condition has primarily been observed in multiparous women with its occurrence linked to trauma caused by repeated pregnancies, abdominal distension, or surgery. Risk factors for PPPXE include obesity, previous abdominal surgery, and large ascites; all of which can contribute to increased mechanical stress on the skin in the periumbilical area.^3^, ^4^, ^5^ In this report, we present a case of PPPXE in an elderly multiparous woman with extensive comorbidities, contributing to the existing body of literature on this extremely rare condition.

## Case report

An 85-year-old woman, gravida 18, presented for evaluation of a pruritic plaque on her abdomen that had been present for 2 years. Her past medical history included chronic kidney disease, hypertension, type 2 diabetes mellitus, myocardial infarction, stroke, bilateral below knee amputation due to osteomyelitis, and diabetes-associated retinopathy. She had abdominal surgery (cholecystectomy) in 2020.

**Fig 1:**
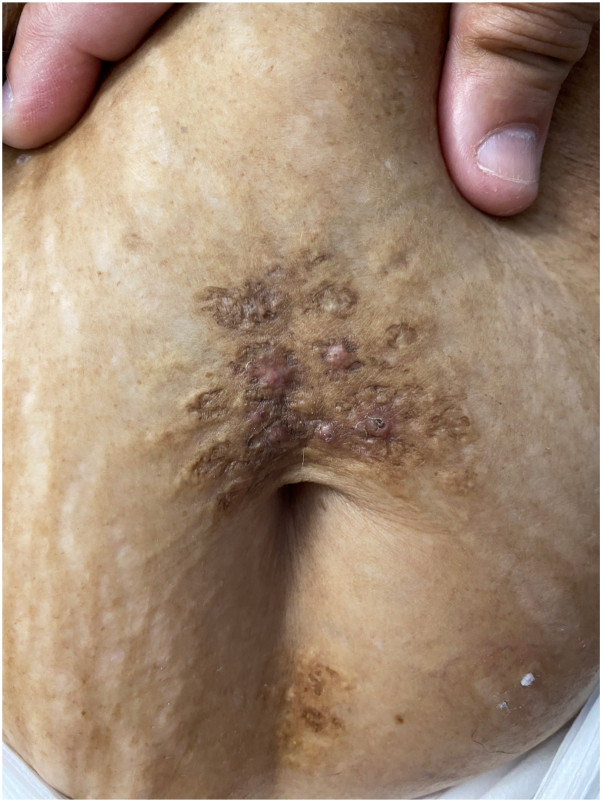
Keratotic, yellow plaques above and below the umbilicus.

On physical examination, there was a keratotic, yellow plaque above her umbilicus with several erythematous papules. A few of the papules had crater-like centers. There was a smaller yellow plaque below her umbilicus (Fig 1).

**Fig 2:**
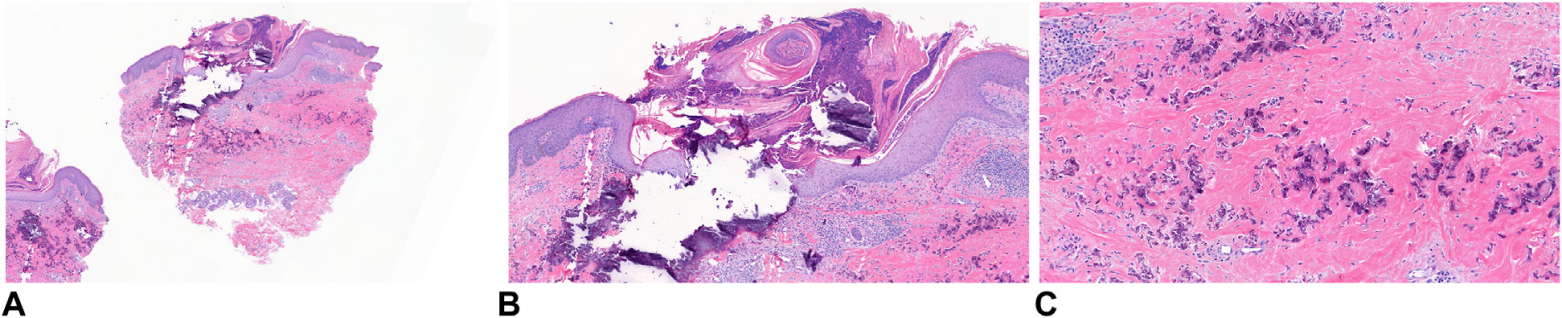
Punch biopsy at 40× magnification (**A**), 100× magnification (**B**) and 200× magnification (**C**). Histologic exam revealed dermal collections of fragmented and irregularly clumped elastic fibers with basophilic staining, consistent with calcification. The perforation characteristic of PPPXE was demonstrated by extrusion of basophilic aggregations of calcified material through the epidermis. *PPPXE*, Periumbilical perforating PXE.

A clinical diagnosis of periumbilical pseudoxanthoma elasticum was made, based on the clinical presentation and the patient’s history of 18 pregnancies. Three 4-mm punch biopsies were performed to confirm the clinical diagnosis. Histologic exam revealed dermal collections of fragmented and irregularly clumped elastic fibers with basophilic staining, consistent with calcification. The histopathology slides show classic fragmented, calcified elastic fibers in the mid-dermis, identical to the findings in PXE. The perforation characteristic of PPPXE was demonstrated by extrusion of basophilic aggregations of calcified material through the epidermis (Fig 2, *A*-*C*).

When the patient returned for suture removal 2 weeks later, she opted for no treatment, since the most protuberant papules had been removed.

## Discussion

The inheritance pattern of PXE remains unclear. PXE demonstrates considerable heterogeneity and variable expression with even intrafamilial variation in phenotype.^1^^,^^6^ For example, Uitto et al describe cases of PXE in families with skin manifestations and very little ophthalmologic or cardiovascular findings, and others with considerable ophthalmologic or cardiovascular findings with little cutaneous findings.^1^

Uitto put forth that intrafamilial variation could be due to metabolic pathways linking mutations in the *ABCC6* gene with the distinct phenotypes in different tissues.^1^ This variability has led researchers to propose the existence of modifying factors, such as additional genetic mutations, environmental influences, or epigenetic changes, that may affect how PXE manifests in different individuals.^1^^,^^6^

In addition to possible genetic predisposition, the mechanical trauma hypothesis remains a widely accepted explanation for the development of PPPXE, especially in the context of repeated pregnancies. The physical stress on the periumbilical skin, combined with hormonal changes affecting connective tissue elasticity, may trigger localized degeneration and calcification of elastic fibers, leading to the formation of PPPXE lesions.^5^ Initially thought to be a distinct entity confined to multiparous women, further cases have been reported of PPPXE occurring in conjunction with PXE in other locations,^5^^,^^7^ suggesting PPPXE may be a variable expression of hereditary PXE. Juhn et al reported a case of a multiparous, gravida 9, patient with PPPXE who also had PXE lesions on her anterior neck.^5^ Kumar et al describe a case of a nulliparous woman with periumbilical PXE in addition to a lesion on her neck.^7^

Kocaturk et al put forth that the presence of PPPXE in patients with chronic renal failure with abnormal phosphate-calcium metabolism may contribute to transepidermal elimination.^3^ Sapadin et al present a case of a patient with chronic renal failure and PPPXE that improved after hemodialysis, suggesting that chronic renal failure may play a role in precipitating PPPXE.^8^

Given the rarity of PPPXE and the challenges in differentiating it from other dermatologic conditions, clinicians should maintain a high index of suspicion when evaluating patients with periumbilical lesions, especially those with a history of multiple pregnancies or relevant comorbidities.

## Conflicts of interest

None disclosed.
